# Integrating CT-based radiomic model with clinical features improves long-term prognostication in high-risk prostate cancer

**DOI:** 10.3389/fonc.2023.1060687

**Published:** 2023-04-27

**Authors:** Jerry C. F. Ching, Saikit Lam, Cody C. H. Lam, Angie O. Y. Lui, Joanne C. K. Kwong, Anson Y. H. Lo, Jason W. H. Chan, Jing Cai, W. S. Leung, Shara W. Y. Lee

**Affiliations:** ^1^Department of Health Technology and Informatics, The Hong Kong Polytechnic University, Hong Kong, Hong Kong SAR, China; ^2^Department of Biomedical Engineering, The Hong Kong Polytechnic University, Hong Kong, Hong Kong SAR, China; ^3^Research Institute for Smart Aging, The Hong Kong Polytechnic University, Hong Kong, Hong Kong SAR, China

**Keywords:** radiomic, high-risk, prostate cancer, prognosis, progression-free survival (PFS), radiation therapy, prostate-only radiotherapy, radiomic-clinical model

## Abstract

**Objective:**

High-risk prostate cancer (PCa) is often treated by prostate-only radiotherapy (PORT) owing to its favourable toxicity profile compared to whole-pelvic radiotherapy. Unfortunately, more than 50% patients still developed disease progression following PORT. Conventional clinical factors may be unable to identify at-risk subgroups in the era of precision medicine. In this study, we aimed to investigate the prognostic value of pre-treatment planning computed tomography (pCT)-based radiomic features and clinical attributes to predict 5-year progression-free survival (PFS) in high-risk PCa patients following PORT.

**Materials and methods:**

A total of 176 biopsy-confirmed PCa patients who were treated at the Hong Kong Princess Margaret Hospital were retrospectively screened for eligibility. Clinical data and pCT of one hundred eligible high-risk PCa patients were analysed. Radiomic features were extracted from the gross-tumour-volume (GTV) with and without applying Laplacian-of-Gaussian (LoG) filter. The entire patient cohort was temporally stratified into a training and an independent validation cohort in a ratio of 3:1. Radiomics (R), clinical (C) and radiomic-clinical (RC) combined models were developed by Ridge regression through 5-fold cross-validation with 100 iterations on the training cohort. A model score was calculated for each model based on the included features. Model classification performance on 5-year PFS was evaluated in the independent validation cohort by average area-under-curve (AUC) of receiver-operating-characteristics (ROC) curve and precision-recall curve (PRC). Delong’s test was used for model comparison.

**Results:**

The RC combined model which contains 6 predictive features (tumour flatness, root-mean-square on fine LoG-filtered image, prostate-specific antigen serum concentration, Gleason score, Roach score and GTV volume) was the best-performing model (AUC = 0.797, 95%CI = 0.768-0.826), which significantly outperformed the R-model (AUC = 0.795, 95%CI = 0.774-0.816) and C-model (AUC = 0.625, 95%CI = 0.585-0.665) in the independent validation cohort. Besides, only the RC model score significantly classified patients in both cohorts into progression and progression-free groups regarding their 5-year PFS (p< 0.05).

**Conclusion:**

Combining pCT-based radiomic and clinical attributes provided superior prognostication value regarding 5-year PFS in high-risk PCa patients following PORT. A large multi-centre study will potentially aid clinicians in implementing personalised treatment for this vulnerable subgroup in the future.

## 1 Introduction

Prostate cancer (PCa) ranks the second highest globally in terms of the prevalence of male malignancies, with more than 1.4 million new cases diagnosed in 2020 (1). High-risk PCa accounts for over one-third of the newly diagnosed PCa population, with a three-fold greater risk of developing distant metastasis compared to their low-risk counterparts. The management strategies of these two cohorts differ drastically. The low-risk PCa usually requires only active surveillance, while high-risk ones require combined modality therapy such as surgery, radiotherapy, systemic chemotherapy or hormonal therapy (2). Optimising treatment strategy for the high-risk is challenging.

Clinically undetectable occult pelvic nodal metastasis is commonly present in up to 40% of high-risk PCa patients (3). However, reliable detection of occult pelvic lymph node (PLN) metastasis is yet available for clinical use (4). It remains as an unresolved clinical challenge as to whether PLN should be prophylactically treated. The survival benefits and toxicity profiles of prostate-only radiotherapy (PORT), or prophylactic whole-pelvic radiotherapy (WPRT) were vigorously investigated in large randomised controlled trials (e.g. RTOG-9413), national database analysis and retrospective studies among the western population (5–9). Hence, the trade-off between better survival with WPRT and reduced toxicities with PORT is still highly debated.

Although the 5-year survival of high-risk PCa patients drastically increased by 23% over the years with greater emphasis on health-related quality of life (HRQoL) (10, 11), over 50% of high-risk PCa patients receiving PORT experienced recurrence, which was far higher than the 12.5% recurrence from the WPRT cohort (6). It is evident that a more refined subgrouping is necessary to predict which high-risk patient receiving PORT would experience recurrence within 5 years to support the clinical decision. Two commonly used conventional risk stratification tools are the National Institute for Health and Care Excellence (NICE) guideline and the Roach formula. The NICE guideline stratified patients into low, intermediate and high risk by clinical (c)T stage, prostate-specific antigen (PSA) serum concentration and Gleason score (GS) (12). Although all are prognostic markers (13–15), the cT stage is determined by digital rectal examination (DRE) that is subjected to high interobserver variability because (16) only a small portion of the prostate is palpable (17). The Roach score (RS) is also commonly used for risk stratification, based on the PSA and GS (18). A score of ≥15% is considered high-risk. A recent study reported that the RS has statistically significantly higher predictive power than the NICE guideline, with a concordance index of 0.724 and 0.715 respectively (13). However, the RS tends to overestimate the risk of occult PLN disease by 2.5 to 4 times among high-risk patients. This would result in the over-treatment of patients and compromise the therapeutic index (19, 20). Therefore, both NICE and RS may not be sufficiently effective in the era of precision medicine. There is a growing demand for developing a more refined, personalised risk stratification method for predicting treatment outcomes of high-risk PCa patients.

Recent advancement in artificial intelligence and radiomics accelerates the development of precision and personalised medicine. Radiomics adopts high-throughput methods to extract high-dimensional radiological features, transforming them into imaging biomarkers to improve clinical decisions (21). Magnetic resonance imaging (MRI) has been extensively studied, employing derived radiomic features for diagnosis or risk prediction of PCa. They showed promising classification performance on clinical endpoints such as GS or biochemical recurrence (BCR) (22–26). By contrast, very few investigations were conducted on the prognostic value of imaging biomarkers derived from the pCT of high-risk PCa patients, despite that the prognostic power of CT-based radiomics has been widely reported in other types of primary solid tumours such as non-small cell lung cancer, nasopharyngeal carcinoma and renal cell carcinoma (27–30). Franzese et al. (31) was the only study employing pCT-derived radiomic features to predict metastasis-free survival in PCa patients treated by external beam radiotherapy (EBRT). However, the included patients in their study were treated with various kinds of treatment, including trans-urethral resection of the prostate, high-intensity focused ultrasound, and EBRT. The prognostic power of their model specifically on PORT-treated high-risk PCa patients remains to be explored.

To our best knowledge, this is the first study to investigate the feasibility of utilising pCT-derived radiomic features and clinical attributes to predict 5-year progression-free survival (PFS) in high-risk localised PCa patients following PORT. Recently, a systematic review in prostate radiomics suggested that incorporating clinical features into a radiomic model may improve its clinical utility (32). Given the inherently heterogeneous nature of the disease, conventional clinical factors may provide additional prognostic value (33). The success of this study may provide insightful findings for clinicians to optimise treatment strategies for managing high-risk PCa patients.

## 2 Methods and materials

### 2.1 Patients

#### 2.1.1 Patient source

**Figure 1:**
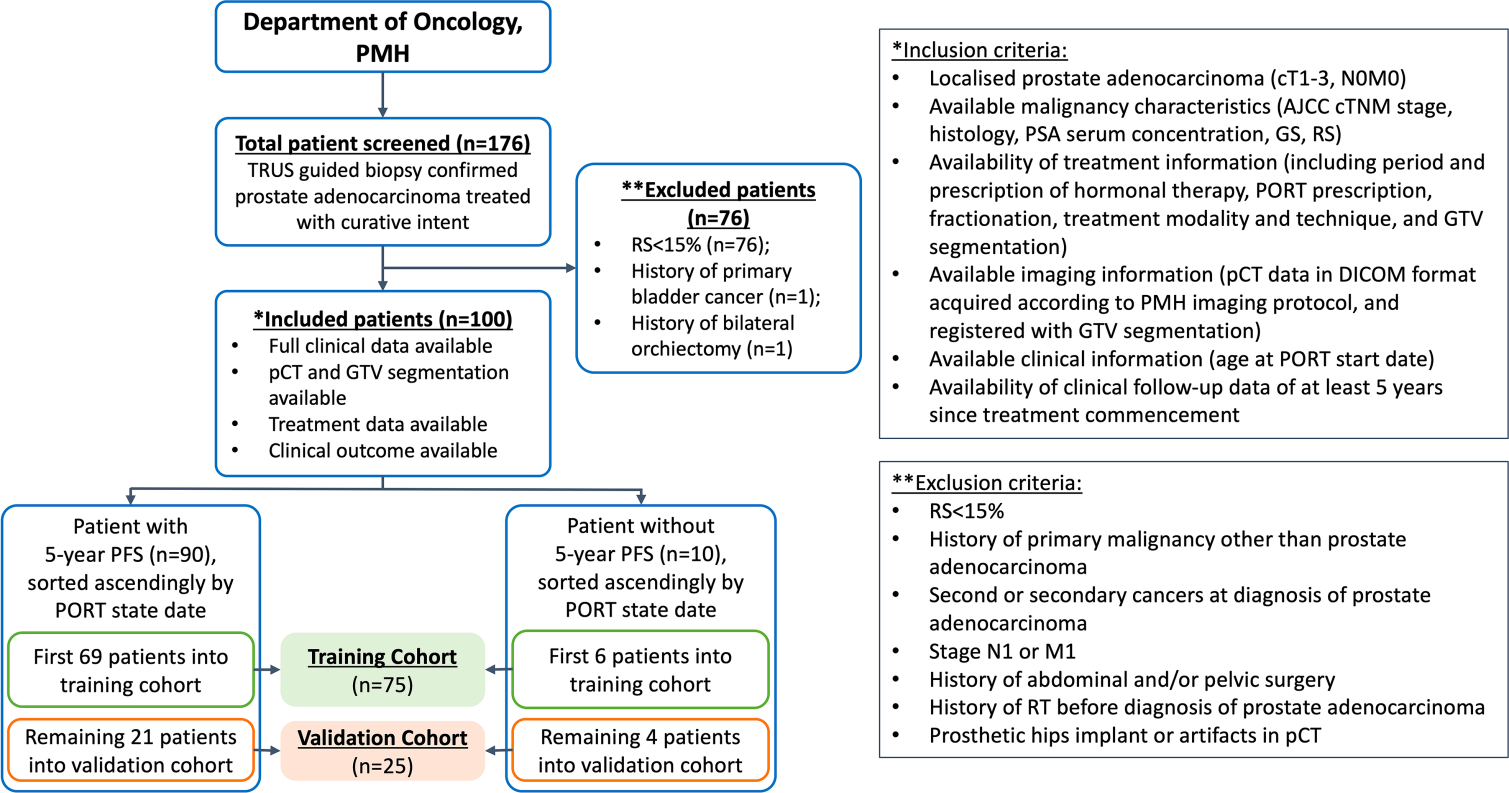
Patient stratification and inclusion-exclusion criteria. PMH, Princess Margaret Hospital; n denotes number of patients; TRUS, transrectal ultrasound; RS, Roach Score; pCT, planning computed tomography; GTV, gross tumour volume; PORT, prostate-only radiotherapy; AJCC, American Joint Committee on Cancer; PSA, prostate specific antigen; GS, Gleason score.

The present study was approved by the Human Subjects Ethics Sub-committee of the Hong Kong Polytechnic University (Reference: HSEARS20220406011) and Kowloon West Cluster Research Ethics Committee (KWC-REC) of the Hong Kong Hospital Authority (Reference: KW/EX-21-155 (165–05)). The requirement for individual informed consent was waived due to the retrospective nature of this study. One hundred and seventy-six biopsy-proven PCa patients who underwent definitive PORT at the Princess Margaret Hospital (PMH) in Hong Kong between February 2011 and December 2016 were retrospectively screened for eligibility. Following the inclusion and exclusion criteria (IEC) shown in **Figure 1**, 100 patients with localised (cT1-3, N0, M0) disease, with RS ≥ 15% (i.e. high-risk PCa) were included in this study.

#### 2.1.2 Patient data

Clinical information such as the age at diagnosis, PORT start date and disease characteristics (clinical tumour (T), nodal (N) and distant metastatic (M) staging, histology, PSA serum concentration, GS, RS); treatment information (prescription and period of PORT and hormonal therapy, treatment techniques and organ contours); imaging information (pCT registered with organ contours); and clinical outcome (status of biochemical failure, regional and distant metastasis) were collected retrospectively.

#### 2.1.3 Treatment approach and clinical endpoint

All patients were treated with PORT using intensity-modulated radiotherapy (IMRT), to a total of 70 or 74Gy. The treatment regimen also included neoadjuvant and concurrent antiandrogen and/or luteinising hormone-releasing hormone analogue (LHRHa) for 8-12 weeks, and 3 years of adjuvant LHRHa.

For this study, the clinical endpoint was the 5-year PFS status. Patients with disease progression manifested as biochemical recurrence, regional or distant metastasis, or death (34) were labelled “PFS-1”, with the others labelled as “PFS-0”. The Phoenix criteria of biochemical recurrence, defined as > 2 ng/mL rise from nadir PSA (35), was adopted in this study.

#### 2.1.4 Patient stratification

The enrolled patients with treatment commenced on or before the date of 08/11/2016 were allocated to a training dataset (n = 75), and the remaining ones were assigned to an independent validation dataset (n = 25). The PFS-1 to PFS-0 ratio between the training and independent validation cohort was set at 6:4, referencing a similar work adopting temporal stratification (36). This patient stratification approach has been widely adopted in similar studies (36–38), which complies with the recommendation provided in the Transparent Reporting of a Multivariable Prediction Model for Individual Prognosis or Diagnosis (TRIPOD) type-2b classification (39).

### 2.2 Planning CT acquisition and volume-of-interest segmentation

**Figure 2:**
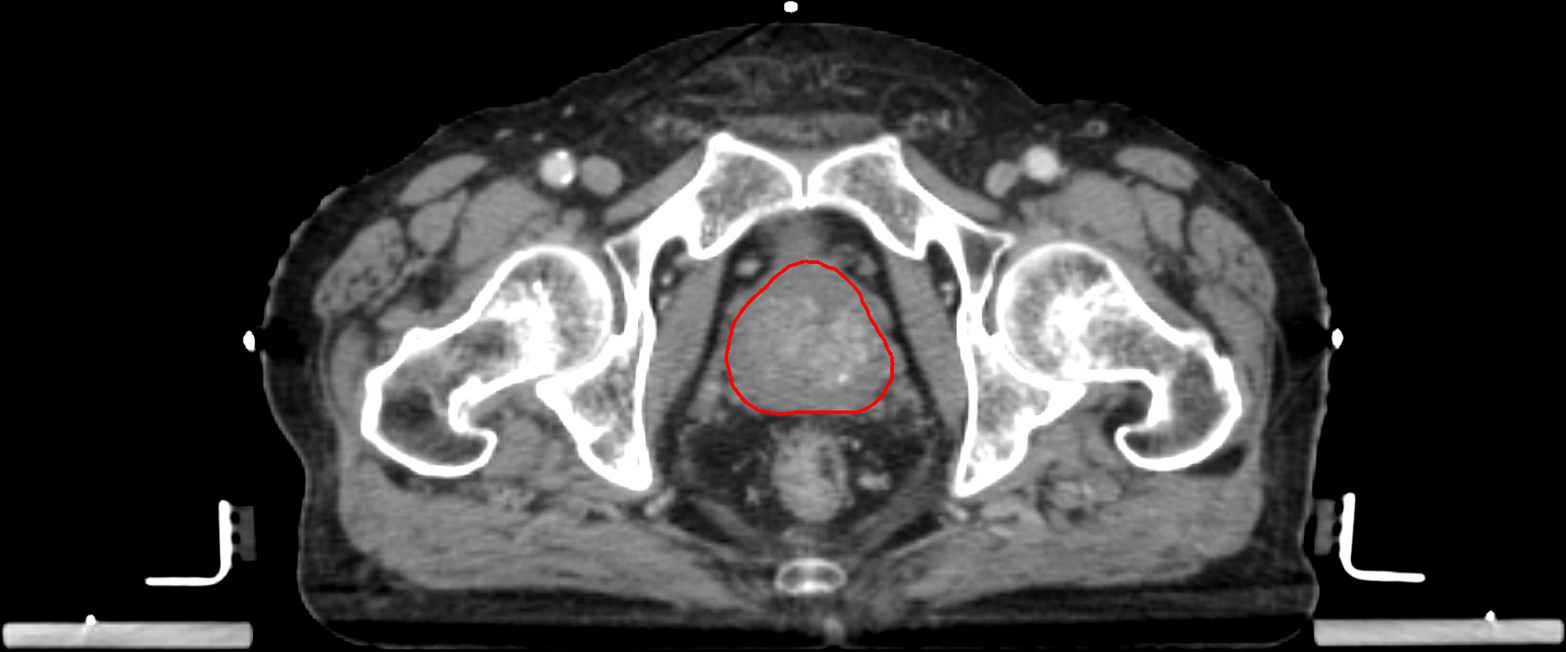
Representative example of a GTV segmentation on contrast-enhanced pCT of a high-risk prostate cancer patient.

Patients underwent iodinated contrast-enhanced (intravenous injection of 120mL Omnipaque 300 mg/dL at 3 mL/second with 75 seconds scan delay) on one of the two CT scanners: 16-slice GE LightSpeed RT16 or GE BrightSpeed (GE Medical Systems, WI, USA). The pCT acquisition parameters included: X-ray tube voltage 120 kVp or 140 kVp; X-ray tube current 114-376 mA; field-of-view 500-650 mm; body filter; standard convolution kernel; matrix size 512x512; pixel spacing 0.98-1.27 mm; and reconstruction thickness 2.5 mm. The pCT scans were acquired 24 (range, 6-47) days on average before PORT commencement. All pCT scans were stored in Picture Archiving and Communication System (PACS) in Digital Imaging and Communications in Medicine (DICOM) format. All pCTs scans were registered with GTV segmentation, which was the VOI for radiomic feature extraction and modelling in this study. The GTV of all patients were segmented by experienced oncologists on the Eclipse ARIA system (version 8.6.15 or 13.0.26, Varian Medical System). The delineation of VOI is performed according to the ESTRO ACROP consensus guideline (40). A team of oncologists with expertise in prostate cancer radiotherapy delineated the ROI. To address the inter-observer variabilities, the contours were all reviewed and approved by a Consultant Oncologist before use. Calcification, necrosis, nor artifacts due to fiducial markers were not found in all the included cases in this study. The GTV segmentation of a representative patient is illustrated in **Figure 2**.

### 2.3 CT image pre-processing

All pCT images were pre-processed before radiomic feature extraction, in compliance with the recommendations provided in the internationally accepted Image Biomarker Standardisation Initiative (IBSI) guideline (41). All steps were implemented by an in-house developed pipeline (IhDP) which used an open-source Pyradiomics v2.2.0 with SimpleITK v1.2.4 package on Python v3.7.3 platform. pCT was first resampled to 1 mm isotropic voxels by B-spline interpolation to account for voxel spacing variation while avoiding longitudinal image oversampling (41). Resampled pCT were discretized to 10-Hounsfield unit (HU) bins to homogenise intensity resolution (42). The GTV was re-segmented by HU thresholding, bounded by -150 and 180 HU (43). Six kernel sizes of Laplacian of Gaussian (LoG) filters, 0.5, 1, 2, 3, 4 and 5 mm, were used to reconstruct the image, facilitating fine and coarse texture feature extraction at different resolutions (44).

### 2.4 Feature extraction & feature normalization

A total of 665 radiomic features from the GTV were extracted using the IhDP. Types of the extracted features of both unfiltered and LoG-filtered images included shape (n = 14), first-order intensity (n = 108) and texture (n = 543). Details of the extracted features classes and their distributions are shown in **Supplementary Material**.

Before analysis, all radiomic features were normalised by using z-score transformation using R software (version 4.1.3). Specifically, features were centred and scaled firstly in the training cohort in which each feature had a mean of 0 and a standard deviation of 1. The normalisation factors obtained in the training cohort were then used to perform feature normalisation in the independent validation cohort.

### 2.5 Feature selection & model development

Relevant feature selection processes including the Spearman correlation coefficient (SCC) and Mann-Whitney U test were performed on the training cohort before the use of Ridge regression algorithms (44–46). The independent validation cohort was adopted for independent model validation. All model training and validation were performed using the R software.

For the radiomic model development, the SCC was first calculated for each pair of radiomic features in the training cohort using the R package “caret”. When the absolute correlation coefficient (r) was ≥ 0.6 in any of the feature pairs, the feature with a higher mean absolute correlation was removed from the original feature set to minimise the likelihood of multicollinearity and model overfitting (21). The clinical significance of the remaining features was assessed by using a two-sided, unpaired Mann-Whitney U test in the training cohort by executing the “wilcox.test’ function in the R software, while features with p > 0.1 were removed. A L2 regularisation was performed using ridge regression in the R package “glmnet”. Ridge regression penalises regression coefficients through hyperparameter (λ) tuning in a grid search. The λ yielding a minimum cross-validation error was then chosen. The objective function was optimised through cyclical coordinate descent in the R package “glmnet”.

For the clinical model development, clinical features including PSA serum concentration, GS, RS and GTV volume formed the initial feature set. PSA was categorised into< 10, 10-20 and > 20 ng/mL while GS was classified into five groups (14). RS was divided into 4 categories: 15-25%, > 25-35%, > 35%-45% and > 45% (18). All clinical features were tested for correlation using the SCC test in the training cohort. The same correlation threshold of r ≥ 0.6 as in the R model was applied. Ridge regression was also performed.

To develop the radiomic-clinical (RC) combined model, all selected radiomic and clinical features were combined and fitted into the ridge regression. A 5-fold cross-validation was employed with 100 iterations to obtain the average predictive performance model. The model score was calculated as


Model Score=(Coefficient)×(Feature Value)+Intercept

Three model scores were calculated for each patient: R (R-score), C (C-score) and RC combined (RC-score) models. The R package “cvAUC” and “PRROC” were used to compute the averaged area under the receiver-operating characteristics (ROC-AUC) curves and precision-recall curve (PRC). Other performance indicators including sensitivity, specificity, accuracy and the Youden index (YI) were calculated by the R package “pROC”. The optimal cut-off in each model was determined using the Youden method (47).

### 2.6 Statistical analysis

All continuous variables have been verified for conformity by the Shapiro-Wilk test. Statistical differences of continuous clinical and demographic variables were evaluated by the Mann-Whitney U test, while categorical variables were assessed by Chi’s square or Fisher-exact test as appropriate. Performance of the R, C and RC combined models were compared using the Delong test, based on their averaged ROC-AUC in both training and independent validation cohorts. The correlations between RC-score, radiomic and clinical variables were evaluated by SCC to reveal any dominant features in the model that contribute heavily to the RC-score. SCC was also used to assess inter-feature correlation in the training cohort for feature screening. All statistical tests were two-sided with a value of p< 0.05 considered statistically significant.

## 3 Results

### 3.1 Patient characteristics

Among 176 patients screened for eligibility, 100 cases met the inclusion criteria. 10 included patients who experienced disease progression were labelled as “PFS-1”, in which 6 (8%) and 4 (16%) were allocated to the training and independent validation cohorts respectively. **Table 1** summarises the characteristics of the enrolled patients.

**Table 1:** Patient characteristics.

Patient Characteristics	Whole Cohort	Training Cohort				Validation Cohort				p**
		All	PFS-1	PFS-0	p*	All	PFS-1	PFS-0	p*	
Patients, n	100	75	6	69	–	25	4	21	–	–
Age at PORT start date, median (range)	72 (52–86)	72 (55-84)	70 (58-78)	72 (55-84)	0.66	70 (52-86)	69 (52-86)	73.5 (68-82)	0.32	0.93
PSA before PORT, ng/mL, n (%)					0.37				0.80	0.11
< 10	10 (10)	6 (8)	0 (0)	6 (8.7)		4 (16)	1 (25)	3 (14.3)		
10-20	29 (29)	19 (25.3)	3 (50)	16 (23.2)		10 (40)	1 (25)	9 (42.9)		
> 20	61 (61)	50 (66.7)	3 (50)	47 (68.1)		11 (44)	2 (50)	9 (42.9)		
GS grade group, n (%)					0.75				0.64	0.11
GS ≤ 6	11 (11)	11 (14.7)	0 (0)	11 (15.9)		0 (0)	0 (0)	0 (0)		
GS = 3 + 4	21 (21)	14 (18.7)	2 (33.3)	12 (17.4)		7 (28)	1 (25)	6 (28.6)		
GS = 4 + 3	17 (17)	13 (17.3)	1 (16.7)	12 (17.4)		4 (16)	0 (0)	4 (19.0)		
GS = 8	24 (24)	20 (26.7)	1 (16.7)	19 (27.5)		4 (16)	0 (0)	4 (19.0)		
GS = 9-10	27 (27)	17 (22.7)	2 (33.3)	15 (21.7)		10 (40)	3 (75)	7 (33.3)		
RS, n (%)					0.94				0.61	0.71
15-25%	29 (29)	22 (29.3)	1 (16.7)	21 (30.4)		7 (28)	0 (0)	7 (33.3)		
> 25-35%	19 (19)	16 (21.3)	2 (33.3)	14 (20.3)		3 (12)	0 (0)	3 (14.3)		
> 35-45%	22 (22)	15 (20)	1 (16.7)	14 (20.3)		7 (28)	2 (50)	5 (23.8)		
> 45%	30 (30)	22 (29.3)	2 (33.3)	20 (29.0)		8 (32)	2 (50)	6 (28.6)		
GTV volume, cm^3^, median (range)	42.7 (15.7-170.6)	43.5 (15.7-170.6)	40.9 (24.9-86.8)	43.5 (15.7-170.6)	0.52	35.6 (18.1-100.4)	34.1 (22.5-63.7)	41.7 (18.1-100.4)	0.92	0.10
PORT dose/fractionation, n (%)					0.29				0.16	< 0.001
70Gy/35fr	31 (31)	31 (41.3)	4 (66.7)	27 (39.1)		0 (0)	0 (0)	0 (0)		
74Gy/35fr	67 (67)	43 (57.3)	2 (33.3)	41 (59.4)		24 (96)	3 (75)	21 (30.4)		
74Gy/37fr	2 (2)	1 (1.3)	0 (0)	1 (1.4)		1 (4)	1 (25)	0 (0)		
NHT, n (%)					> 0.99				0.42	0.18
Antiandrogen only	4 (4)	2 (2.7)	0 (0)	2 (2.9)		2 (8)	1 (25)	1 (4.8)		
Antiandrogen with LHRHa	94 (94)	72 (96)	6 (100)	66 (95.7)		22 (88)	3 (75)	19 (90.5)		
LHRHa only	1 (1)	0 (0)	0 (0)	0 (0)		1 (4)	0 (0)	1 (4.8)		
None	1 (1)	1 (1.3)	0 (0)	1 (1.4)		0 (0)	0 (0)	0 (0)		
CHT, n (%)					0.71				0.003	0.88
Antiandrogen only	18 (18)	12 (16)	1 (16.7)	11 (15.9)		6 (24)	3 (75)	3 (14.3)		
Antiandrogen with LHRHa	59 (59)	45 (60)	5 (83.3)	40 (58)		14 (56)	0 (0)	14 (66.7)		
LHRHa only	18 (18)	14 (18.7)	0 (0)	14 (20.3)		4 (16)	0 (0)	4 (19)		
None	5 (5)	4 (5.3)	0 (0)	4 (5.8)		1 (4)	1 (25)	0 (0)		
AHT, n (%)					0.11				0.11	0.80
Antiandrogen only	2 (2)	1 (1.3)	0 (0)	1 (1.4)		1 (4)	1 (25)	0 (0)		
Antiandrogen with LHRHa	7 (7)	5 (6.7)	1 (16.7)	4 (5.8)		2 (8)	1 (25)	1 (4.8)		
LHRHa only	81 (81)	61 (81.3)	3 (50)	58 (84.1)		20 (80)	2 (50)	18 (85.7)		
None	10 (10)	8 (10.7)	2 (33.3)	6 (8.7)		2 (8)	0 (0)	2 (9.5)		

PORT, prostate-only radiotherapy; PSA, prostate specific antigen; GS, Gleason score; RS, Roach score; GTV, gross tumour volume; NHT, neoadjuvant hormonal therapy; CHT, concurrent hormonal therapy; AHT, adjuvant hormonal therapy; LHRHa, luteinizing hormone-releasing hormone analogue; n denotes the number of patients; PFS, progression-free survival, *refers to p derived from univariate analysis on association of each patient characteristics with the status of 5-year PFS, **refers to p derived from patient characteristics comparison between the training and validation cohorts.

No statistically significant difference was detected in the distribution of age, PSA serum concentration, GS, RS, GTV volume, neoadjuvant hormonal therapy (NHT), concurrent hormonal therapy (CHT) and adjuvant hormonal therapy (AHT) regimen between training and independent validation cohorts, except for the PORT dose scheme (p< 0.001). Furthermore, PSA, GS, RS and GTV volumes were not significantly different between PFS-1 and PFS-0 patients in both cohorts, except for the CHT regime.

### 3.2 Model development

**Figure 3:**
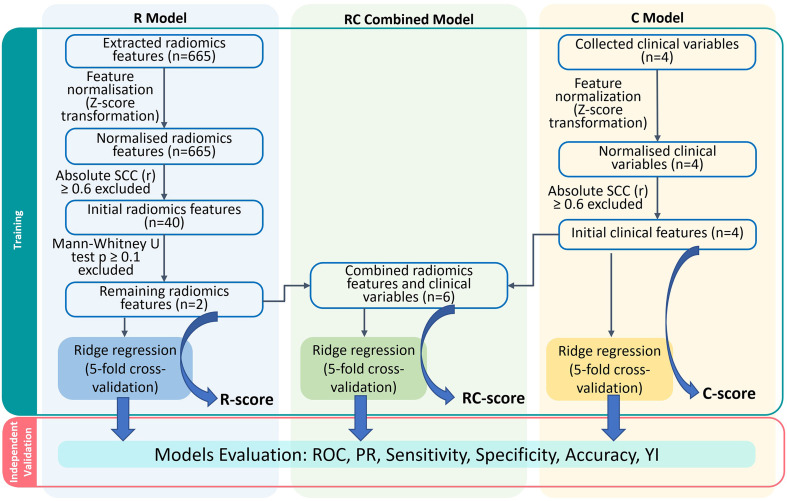
Models construction workflow. R, Radiomics; RC, Radiomic-clinical; C, clinical; n denotes number of features; ROC, receiver-operating characteristic; PR, precision-recall; YI, Youden index.

A simplified modelling workflow is illustrated in **Figure 3**. In the R model, 40 features with high independence (r< 0.6) shown in the correlation map (**Supplementary Material**) underwent further elimination. Among them, the unfiltered-shape-flatness and LoG-1mm-filtered root-mean-square (RMS) features were chosen for the development of the R model. In the C model, the Spearman correlations between PSA, GS, RS and GTV volume were less than 0.6 (**Supplementary Material**). Therefore, all 4 clinical features were used for modelling. The R and C models consisted of 2 radiomic and 4 clinical features respectively, while these 6 features were combined in the RC model.

**Table 2** lists the intercepts and coefficients of all three models. Patients with model scores higher than the optimal cut-off were classified as high-risk of having disease progression within 5 years since the commencement of treatment, or vice versa.

**Table 2:** Intercepts and selected radiomic and/or clinical features of the R, C and RC combined models.

Intercept and Coefficients	Values of Intercept and Coefficients of Each Model		
	R Model	C Model	RC Combined Model
Intercept	-2.463	-2.450	-2.445
Raw_shape_flatness	-0.052	—	-0.010
LoG_1mm_first-order_root-mean-square	-0.101	—	-0.017
PSA	—	-0.032	-0.005
GS	—	0.015	0.004
RS	—	0.033	0.004
GTV volume	—	-0.044	-0.006

R, radiomics; C, clinical; RC, radiomic-clinical; LoG, Laplacian of Gaussian; PSA, prostate specific antigen; GS, Gleason score; GTV, gross tumour volume. —, feature absent.

### 3.3 Model evaluation

**Figure 4:**
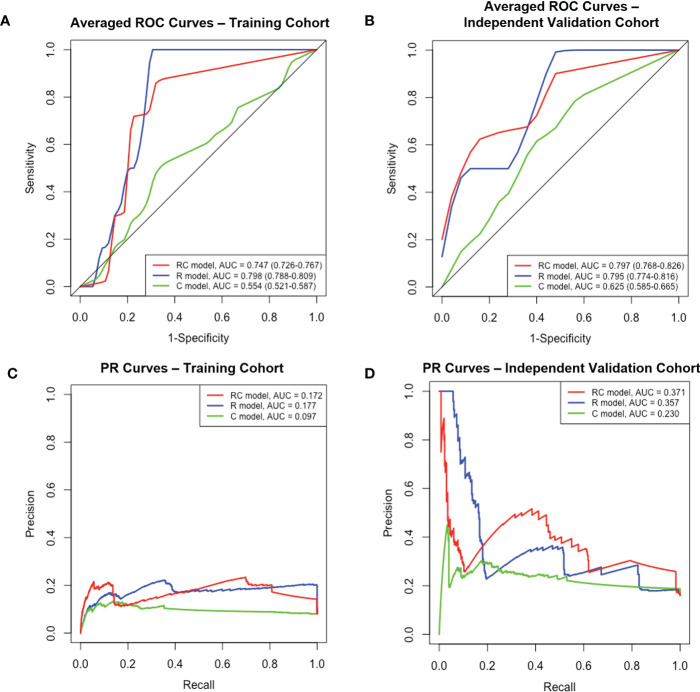
The ROC and PR curves of R, C and RC combined models. Boxplots **(A, B)** Averaged ROC curves of the training and independent validation cohort. Boxplots **(C, D)** The PR curves of both cohorts. ROC, receiver-operating characteristic; PR, precision-recall; R, radiomic; C, clinical; RC, radiomic-clinical; AUC, area under curve.

**Figure 5:**
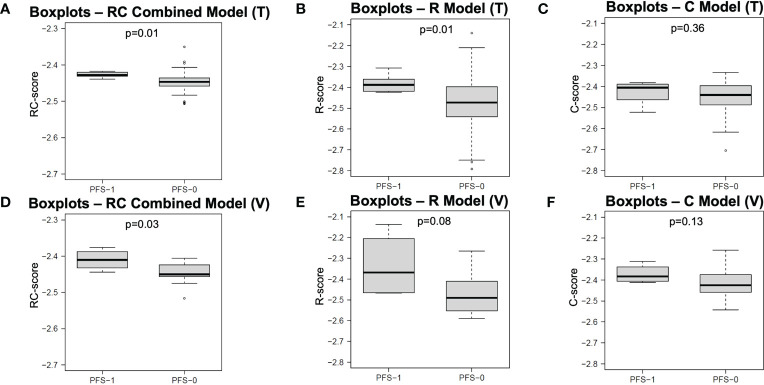
Boxplots of model scores of R, C and RC combined models. The boxplots in first **(A–C)** and second rows **(D–F)** display the distribution of model scores calculated by different models in training and validation cohorts, respectively. R, Radiomics; C, clinical; RC, Radiomic-clinical; T, training cohort; V, validation cohort; PFS, progression-free survival.

The performance of each model in both the training and independent validation cohorts is summarised in **Table 3**. The ROC and PR curves are shown in **Figure 4**. Boxplots of the model scores are presented in **Figure 5**.

**Table 3:** Predictive performance of the R, C and RC combined models.

Model	Training Cohort								Validation Cohort						
	ROC-AUC, Mean (95%CI)	PR-AUC, Mean		Sens.	Spec.	Acc.	YI	p*	ROC-AUC, Mean (95%CI)	PR-AUC, Mean	Sens.	Spec.	Acc.	YI	p*
R	0.798 (0.788-0.809)	0.177		0.998	0.658	0.685	0.656	0.005	0.795 (0.774-0.816)	0.357	0.825	0.605	0.640	0.430	< 0.001
C	0.554 (0.521-0.587)	0.097		0.353	0.764	0.731	0.117	< 0.001	0.625 (0.585-0.665)	0.230	0.530	0.676	0.652	0.206	< 0.001
RC combined	0.747 (0.726-0.767)	0.172		0.808	0.722	0.729	0.530	(Ref.)	0.797 (0.768-0.826)	0.371	0.793	0.653	0.676	0.446	(Ref.)

R, radiomics; C, clinical; RC, radiomic-clinical; ROC, receiver-operating characteristic; AUC, area-under-curve; CI, confidence interval; PR, precision-recall; YI, Youden index (Sensitivity + specificity -1); Sens., sensitivity; Spec., specificity; Acc., accuracy; Ref., reference, *refers to statistical significance of ROC-AUC differences between R and RC combined model and that between C and RC combined model in each cohort

The RC combined model yielded the highest ROC-AUC (0.797, 95%CI = 0.786-0.826) in the independent validation cohort compared to the R (0.795, 95%CI = 0.774-0.816) and C (0.625, 95%CI = 0.585-0.665) models. The DeLong test showed that the RC combined model had a ROC-AUC significantly higher than the C model in both training (0.747 vs. 0.554, p< 0.001) and independent validation (0.797 vs. 0.625, p< 0.001) cohorts. A similar finding was also observed with the RC model demonstrating a higher ROC-AUC than the R model in the independent validation cohort (0.797 vs. 0.795, p<.001). Moreover, the RC combined model had the highest PR-AUC, accuracy and YI compared to R and C models in the independent validation cohort. The RC combined model also attained a relatively high sensitivity and specificity of 0.808 and 0.722 in the training cohort and 0.793 and 0.653 in the independent validation cohort, respectively.

Of note, the RC combined model was the only model that resulted in a significantly different RC-score between PFS-1 and PFS-0 patients in both the training (median: -2.428 vs. -2.447, p = 0.01) and independent validation cohorts (median: -2.411 vs. -2.451, p = 0.03). On the other hand, the R model failed to render a significantly different R-score in the independent validation cohort (median: -2.368 vs. -2.491, p = 0.08). Similarly, the C model did not yield any significant difference in C-score for either the training (median: -2.405 vs. -2.440) or independent validation cohort (-2.383 vs. -2.425).

### 3.4 Correlation analysis among RC-scores, radiomic and clinical features

The average absolute SCC (r) of R features with RC-scores (r = 0.603) was ~2.6 times higher than that of C features with RC-scores (r = 0.235). Among radiomic features, LoG-1mm-filtered RMS had the highest correlation with the RC-score (r = 0.797), followed by the unfiltered-shape-flatness (r = 0.409). The clinical features with the highest and lowest correlation with RC-score were GS (r = 0.411) and GTV volume (r = 0.105) respectively. Between radiomic and clinical features, unfiltered-shape-flatness and LoG-1mm-filtered RMS had the highest correlation with RS (r = 0.372) and GTV volume (r = 0.228), respectively. The correlation matrix (**Supplementary Material**) described in detail the correlation between RC-scores of patients in the independent validation cohort and their corresponding R and C features.

## 4 Discussion

For the first time, we demonstrated that combining CT-based radiomic (unfiltered-shape-flatness, LoG-1mm-filtered RMS) and clinical attributes (PSA, GS group, RS group and GTV volume) provided superior prognostic value for 5-year PFS in high-risk PCa patients following PORT. The DeLong test revealed that the ROC-AUC of the RC combined model was significantly higher than those of the R and C models in the independent validation cohort. The RC model had the highest overall accuracy and YI. Only the RC model score significantly classified patients into “progression” (PFS-1) and “progression-free” (PFS-0) groups according to their 5-year PFS in both training and independent validation cohorts (both p< 0.05). These findings suggested the potential of the RC combined model in supporting clinicians to implement personalized treatment for this vulnerable patient subgroup in the future. For instance, if a patient is classified into the “progression” (PFS-1) group ahead of the commencement of the PORT treatment, clinicians may consider a more aggressive therapy (e.g., WPRT) for improving the prognosis of the given patient.

The two identified radiomic features (unfiltered-shape-flatness and LoG-1mm-filtered RMS) are in line with previous studies using CT images (27, 28, 48, 49). Unfiltered-shape-flatness is a shape feature calculated by the square root of the least axis length divided by the major axis length (48). A value closer to zero indicates the tumour is flatter. This feature was reported in another CT radiomic model for predicting tumour response in lung cancer patients receiving EBRT (49). Additionally, the RC-score was negatively correlated to the flatness, with a lower flatness value indicating a poorer prognosis. Such a finding is consistent with Khodabakhshi’s study, which predicted the OS of patients with renal cell carcinoma (27). The feature LoG-1 mm-filtered RMS is calculated by the mean of all squared intensity values in LoG-1mm-filtered ROI (48). Similar to flatness, it is negatively correlated to the RC-score. The RMS has been reported in a CT radiomic model study for lung adenocarcinoma, in which a lower value was associated with poorer PFS and OS (28). The prognostic implications of these features have not been reported for PCa, and the association between the selected pCT radiomic features and biological properties remains unclear. Indeed, the RC model was dominated by the R features since the absolute SCC of R features with RC-score was ~2.6 times higher than those of clinical features (**Supplementary Material**). Further investigation is needed to explore the biological mechanism of radiomic features.

Another notable finding is the lack of textural features in the RC combined model. It may be attributed to the intrinsic property of pCT images and the clinical endpoint of our study. Most prostate radiomic studies use MRI-derived features for prognostic prediction (22–24, 50). In a study using T2-weighted MRI, features with the highest predictive value originated from the gray-level run-length matrix (GLRLM) texture feature class (23). Another two studies using apparent diffusion coefficient (ADC) MRI for radiomics modelling (23, 24) suggested that the gray-level co-occurrence matrix (GLCM) texture feature class contained the most predictive features. However, in a prostate radiomic study (51) exploring interfraction cone-beam CT, both shape and first-order features have excellent capability in predicting patient outcomes, which was similar to our study. In addition, these two features were also found to be capable of predicting PFS in both nasopharyngeal carcinoma (NPC) and NSCLC (29, 30). Meanwhile, texture features dominate in MRI-based NPC radiomic studies (52, 53). These observations suggested that the feature class selected for PFS prediction could be influenced by the choice of imaging modality. This may be explained by the inherently lower soft-tissue contrast characteristic in CT than in MRI, resulting in less prominent texture features that may be relevant to the PFS. Texture features in CT and MRI have been regarded as the manifestation of tumour heterogeneity. Hence, the dominant feature class may vary according to different clinical endpoints. For instance, texture features are often selected in both CT and MRI prostate radiomic studies when GS is chosen as the clinical endpoint (25, 42). It is not surprising since GS is the gold standard for characterising prostate heterogeneity, while texture features measure the same physical property. The current study demonstrated that both shape (unfiltered-shape-flatness) and first-order features (LoG-1mm-filtered RMS) have similar predictive performance compared to MRI radiomic studies (23, 24, 50). Nonetheless, a further study involving a larger external validation cohort is needed to validate the performance of these two feature classes in predicting the PFS of high-risk PCa patients.

The C model constructed based on clinical factors had the poorest performance. Univariate analysis did not show any statistical significance of these clinical features between PFS-1 and PFS-0 patients in both cohorts. The most probable explanation is that these clinical features are homogeneous within the high-risk subgroup of PCa patients (32). Thus, using the C model alone would lead to the poorest prediction performance. Statistically, the C model also failed to differentiate the 5-year PFS status in both cohorts. These findings are in line with those reported by Fernandes et al. (23) and Bourbonne et al. (26), who have also incorporated PSA and GS in their clinical models. They also retrospectively investigated the clinical predictive model for high-risk PCa patients treated by EBRT (23) and radical prostatectomy during a 5-year follow-up (26). The sensitivity and specificity of the C model were also comparable to those reported by Bourbonne et al. (26) (Sensitivity: 0.53 vs 0.68; Specificity: 0.53 vs. 0.59). These clinical features, however, are not without predictive values when combined with the R model. It is demonstrated by the increase in overall accuracy in both the training and independent validation cohorts after combining R with C models. The potential complementary role between R and C features for prognosis warrants future investigation (54).

Overall, this study explored the potential of combining pCT-derived radiomic and clinical features in the prognostic prediction of high-risk PCa patients receiving PORT. Our study has demonstrated that the classification performance of the combined RC model was comparable to the combined MRI-derived radiomic and clinical models. Among all included features, the shape and first-order features are considered more intuitive than other complex features when interpreting the classifier in predicting 5-year PFS of high-risk patients in the clinical setting (51). Our patient stratification methodology is highly standardised by adopting a temporal validation as in type-2b study according to the TRIPOD guideline (39). Moreover, the use of pCT is preferable due to its higher standardisation, repeatability and calibration of CT over diagnostic MRI (33). For instance, HU in pCT directly quantifies the electron density of the tissue while the pixel value in MR is arbitrarily allocated.

This study has several limitations. First, the sample size is relatively small. This can be attributed to the strict inclusion and exclusion criteria enforced to ensure high-quality radiomics and clinical data. Nonetheless, numerous studies have also contributed insightful findings to the community with a similar sample size as our work (32, 55–57). Second, this work only performed modelling based on data from a single centre as COVID-19 has restricted the research team from conducting data collection from multiple centres. Third, owing to the small sample size and temporal stratification, a class imbalance exists in which only 10% are PFS-1 patients. Although a similar situation was observed in Bosetti’s (2020) work analysing cone-beam CT performance for predicting prostate cancer clinical progression, further study with a larger sample size and proportion of PFS-1 patients would be beneficial to minimise modelling and evaluation bias. Minority class-boosting techniques should also be implemented if appropriate. Fourth, an external testing set was not employed. This would be essential to demonstrate satisfying model generalisability in a multi-centre study before actual clinical application (58). Moreover, Ridge regression, which was a relatively straightforward modelling strategy, was adopted in this work for demonstrating the proposed feasibility of using radiomic-clincial factors to predict patient outcomes. To render the model fit for clinical application, more robust modelling methodologies such as non-linear machine learning techniques and random oversampling should be incorporated when processing multi-centre data sets.

## 5 Conclusion

This study demonstrated the feasibility and potential of using pCT-derived radiomic and clinical features for predicting 5-year PFS in high-risk PCa patients receiving PORT, which is an important clinical research gap that previously lacks investigation The RC combined model provided statistically superior predictive performance than both R and C models in the independent temporal validation cohort. These findings lay the ground for the future development of a combined radiomic-clinical model involving robust modelling techniques, multicentre data and external validation. We hope that this work will bring attention to the academic community and encourage future work to address this on a larger scale towards clinical implementation. Ultimately, it could potentially act as a supportive decision tool predicting the outcome of different treatment regimens to facilitate personalised management of high-risk PCa patients.

## Data availability statement

The raw data supporting the conclusions of this article will be made available by the authors, without undue reservation.

## Ethics statement

The studies involving human participants were reviewed and approved by Human Subjects Ethics Sub-committee of The Hong Kong Polytechnic University and Kowloon West Cluster Research Ethics Committee of the Hong Kong Hospital Authority. Written informed consent for participation was not required for this study in accordance with the national legislation and the institutional requirements.

## Author contributions

JCC, SL, WSL and SWL contributed to the study design, methodology development, results interpretation, and manuscript review. WL offered administrative and material support for data collection. JCC, AOL, and JCK collected patients’ clinical and imaging data. JCC, SL and AOL contributed to image pre-processing and feature extraction. JCC, SL, CCL, AOL and JCK constructed the models. JCC wrote the manuscript. WSL co-supervised the study. SWL supervised the study. All authors contributed to the article and approved the submitted version.
