# Supplementary material for: Integrating CT-based radiomic model with clinical features improves long-term prognostication in high-risk prostate cancer
Source: Front Oncol. 2023 Apr 27;13:1060687. doi: 10.3389/fonc.2023.1060687 (PMC10186349; doi:10.3389/fonc.2023.1060687)
Supplement: Supplementary file 1 [file DataSheet_1.docx]

Supplementary Material

# Classes of radiomics features extracted

Supplementary Table 1 and Figure 1 shows the detailed distribution of extracted features.

**Supplementary Table 1.** Distributions of radiomics feature extracted in the current work.

| Feature Type | Source and Distribution of Extracted Features (n=665) | | | | | | |
| --- | --- | --- | --- | --- | --- | --- | --- |
|  | Raw image | LoG  (σ = 0.5) | LoG  (σ = 1.0) | LoG  (σ = 2.0) | LoG  (σ = 3.0) | LoG  (σ = 4.0) | LoG  (σ = 5.0) |
| *Shape* | 14 | 0 | 0 | 0 | 0 | 0 | 0 |
| *First-order* | 18 | 18 | 18 | 18 | 18 | 18 | 18 |
| *GLRLM* | 16 | 16 | 16 | 16 | 16 | 16 | 16 |
| *GLCM* | 24 | 24 | 24 | 24 | 24 | 24 | 24 |
| *GLDM* | 14 | 14 | 14 | 14 | 14 | 14 | 14 |
| *GLSZM* | 16 | 16 | 16 | 16 | 16 | 16 | 16 |
| *NGTDM* | 5 | 5 | 5 | 5 | 5 | 5 | 5 |
| Sub-total | 107 | 93 | 93 | 93 | 93 | 93 | 93 |

Abbreviations: LoG = Laplacian of Gaussian, GLRLM = gray-level run-length matrix, GLCM = gray-level co-occurrence matrix, GLDM = gray level dependence matrix, GLSZM = gray level size zone matrix, NGTDM = neighbouring gray tone difference matrix, σ denotes kernel size of LoG filter in mm, n denotes number of features


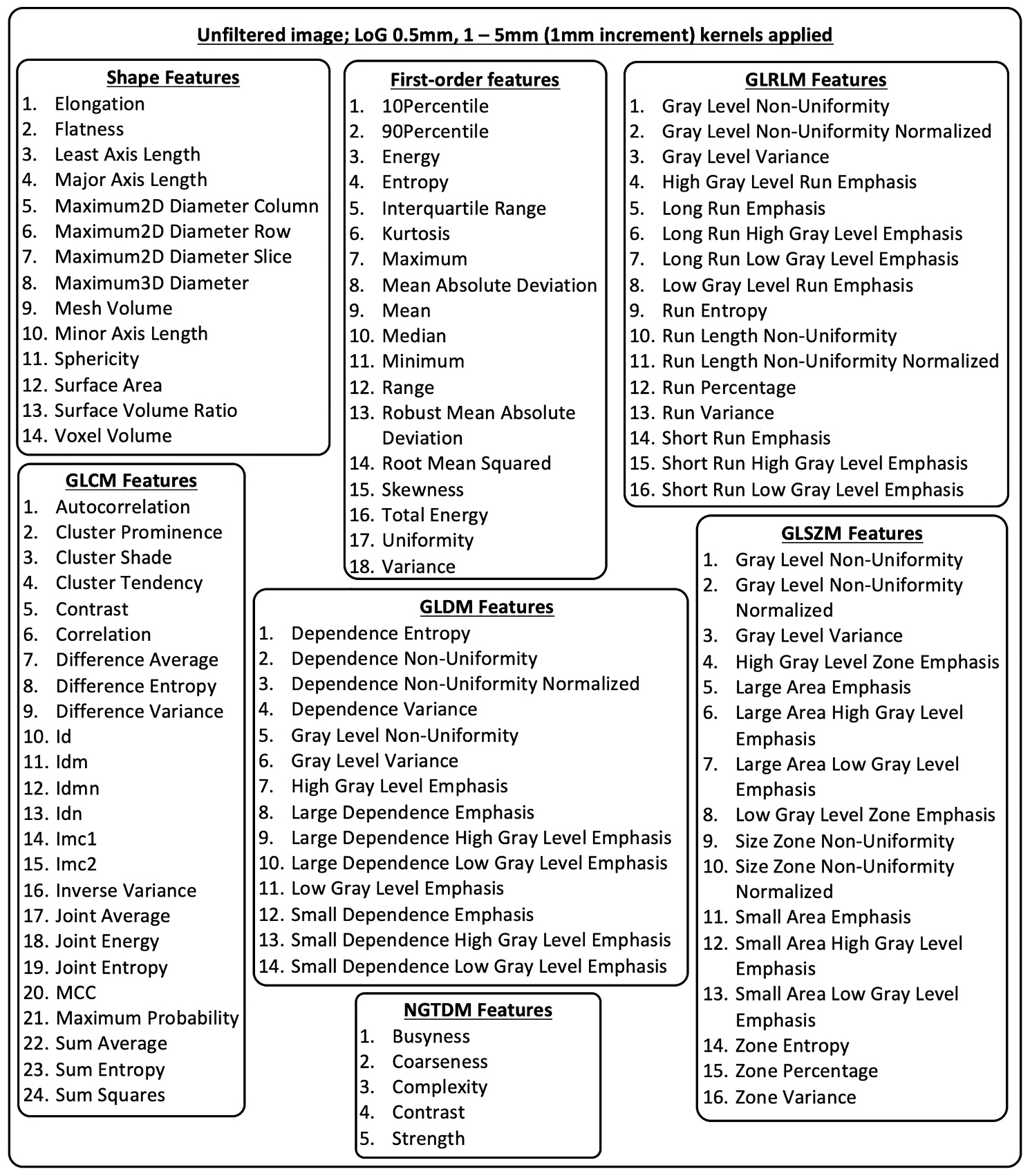


**Supplementary Figure 1.** Detailed list of extracted radiomics feature classes in the current work.

# Classes of radiomics features extracted

Supplementary Figure 2 and Figure 3 shows the correlation between radiomics and clinical features respectively.


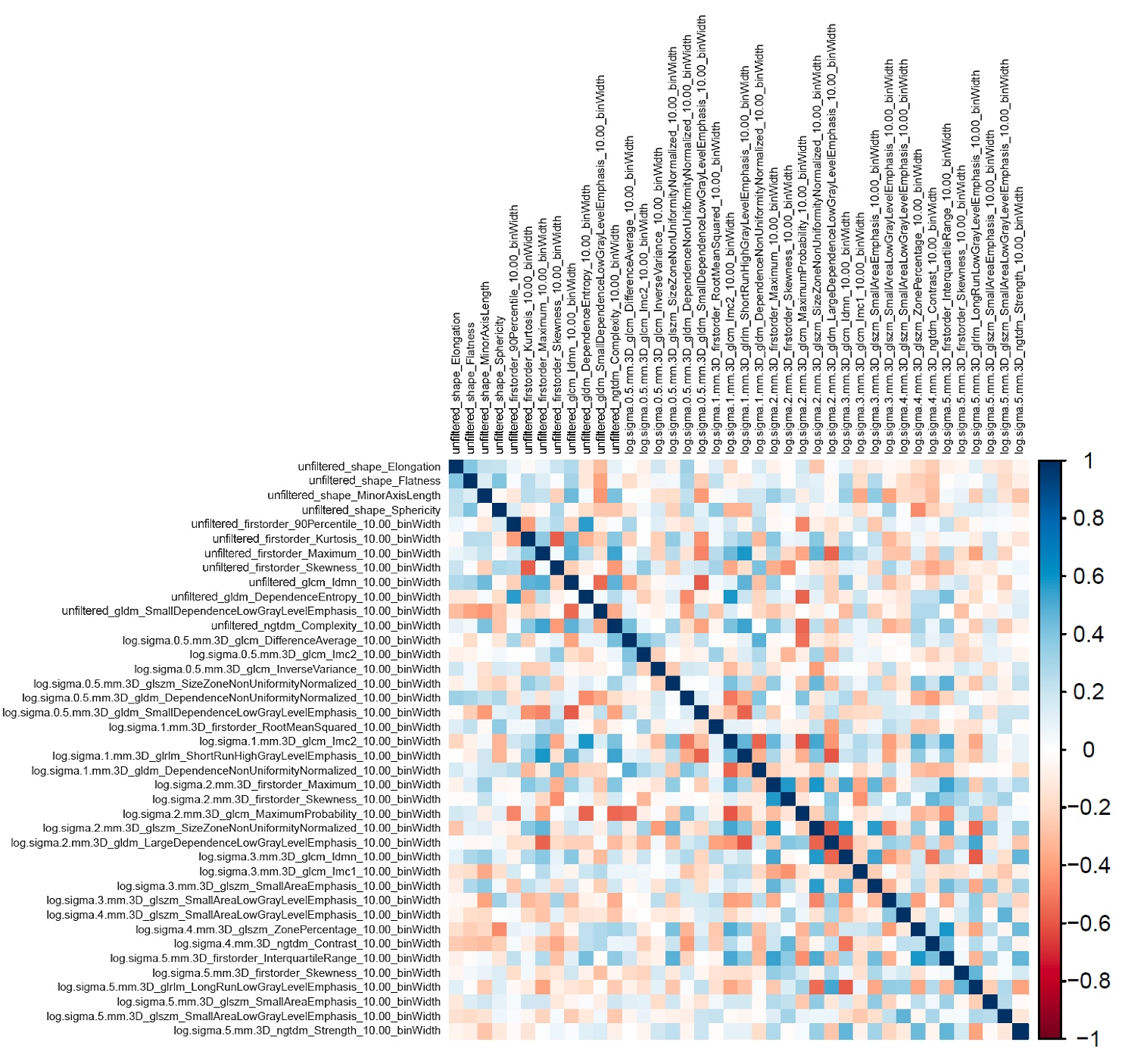


**Supplementary Figure 2.** Correlation matrix of 40 radiomics features with SCC<0.6. Abbreviations: GLCM = gray-level co-occurrence matrix, GLDM = gray level dependence matrix, NGTDM = neighbouring gray tone difference matrix, NGTDM = neighbouring gray tone difference matrix, GLSZM = gray level size zone matrix


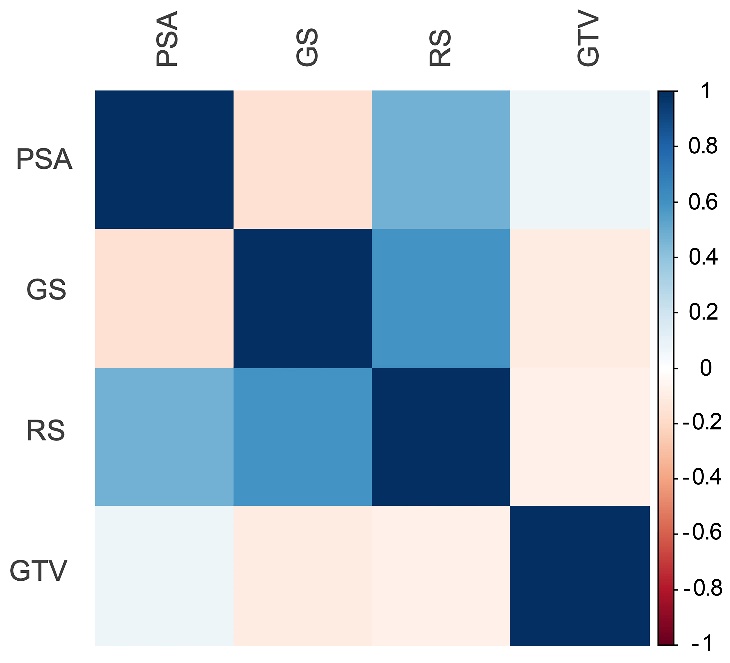


**Supplementary Figure 3.** Correlation matrix of clinical features. Abbreviations: PSA = prostate specific antigen, GS = Gleason score, RS = Roach score, GTV = gross tumour volume

# Correlation analysis of model scores with radiomics and clinical features

Supplementary Figure 4 shows the correlation between the RC scores and both radiomics and clinical features.


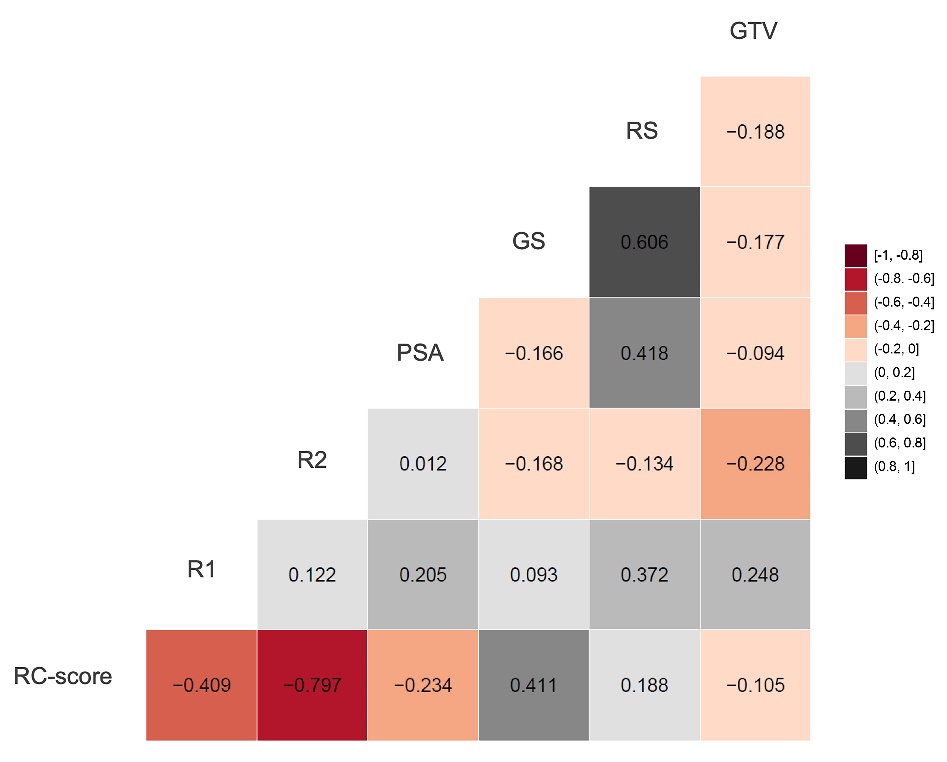


**Supplementary Figure 4.** Correlation matrix of RC score (validation cohort) with radiomics and clinical features. Abbreviations: R1 = Raw_shape_flatness, R2 = LoG_1mm_first-order_root-mean-square, PSA = prostate specific antigen, GS = Gleason score, RS = Roach score, GTV = gross tumour volume.
